# Qualitative translation of relations from BioPAX to SBML qual

**DOI:** 10.1093/bioinformatics/bts508

**Published:** 2012-08-24

**Authors:** Finja Büchel, Clemens Wrzodek, Florian Mittag, Andreas Dräger, Johannes Eichner, Nicolas Rodriguez, Nicolas Le Novère, Andreas Zell

**Affiliations:** ^1^Center for Bioinformatics Tuebingen (ZBIT), University of Tuebingen, 72076 Tübingen, Germany and ^2^European Bioinformatics Institute, Wellcome Trust Genome Campus, Hinxton, Cambridgeshire CB10 1SD, UK

## Abstract

**Motivation:** The biological pathway exchange language (BioPAX) and the systems biology markup language (SBML) belong to the most popular modeling and data exchange languages in systems biology. The focus of SBML is quantitative modeling and dynamic simulation of models, whereas the BioPAX specification concentrates mainly on visualization and qualitative analysis of pathway maps. BioPAX describes reactions and relations. In contrast, SBML core exclusively describes quantitative processes such as reactions. With the SBML qualitative models extension (qual), it has recently also become possible to describe relations in SBML. Before the development of SBML qual, relations could not be properly translated into SBML. Until now, there exists no BioPAX to SBML converter that is fully capable of translating both reactions and relations.

**Results:** The entire nature pathway interaction database has been converted from BioPAX (Level 2 and Level 3) into SBML (Level 3 Version 1) including both reactions and relations by using the new qual extension package. Additionally, we present the new webtool BioPAX2SBML for further BioPAX to SBML conversions. Compared with previous conversion tools, BioPAX2SBML is more comprehensive, more robust and more exact.

**Availability:** BioPAX2SBML is freely available at http://webservices.cs.uni-tuebingen.de/ and the complete collection of the PID models is available at http://www.cogsys.cs.uni-tuebingen.de/downloads/Qualitative-Models/.

**Contact:** finja.buechel@uni-tuebingen.de

**Supplementary Information:** Supplementary data are available at *Bioinformatics* online.

## 1 INTRODUCTION

The goal of systems biology is the model-driven understanding of biological and biochemical processes across all layers and various levels of detail. The biological pathway exchange language (BioPAX) and the systems biology markup language (SBML) are common modeling languages that facilitate the exchange and storage of *in silico* models. BioPAX can be used to describe the biological semantics of metabolic, signaling, molecular, gene-regulatory and genetic interaction networks. It is mainly used for qualitative analysis and information exchange (Demir *et al.*, 2010). SBML describes the structure of models. In contrast to BioPAX, it offers the possibility to include mathematical expressions, which are necessary for dynamic simulations (Hucka *et al.*, 2003). A detailed comparison between both languages has been given by Strömbäck *et al.* (2005, 2006).

Besides these language differences, BioPAX models from databases like the nature pathway interaction database (PID, Schaefer *et al.*, 2009) or MetaCyc (Caspi *et al.*, 2012) are often used as information source to build SBML models for further simulation processes (Bauer-Mehren *et al.*, 2009). The SBML core specification defines quantitative processes, such as reactions, events, rules and constraints, in detail but no other relationships between molecules. Those relationships are denoted as *relations* that specify enzyme–enzyme relations, protein–protein interactions, interactions of transcription factors and genes, protein–compound interaction, links to other pathways, etc. These relations are also provided in BioPAX models, but before the creation of the Qualitative Models extension for SBML (qual, see Berenguier *et al.*, 2011), it was not possible to define those relations or to include reactions together with relations in one model.Hence, new BioPAX to SBML converters are needed.

Today, there exist mainly converters from SBML to BioPAX like *The System Biology Format Converter* (see European Bioinformatics Institute—Computational Systems Neurobiology Group, 2011), but no converter for BioPAX to SBML that is capable of properly including relations. Other research groups previously faced the same problem with incompatibilities between BioPAX and SBML. To overcome the limitations of those file formats and to avoid the creation of pseudo-reactions or similar constructions, Rübenacker *et al.* (2009) introduce an intermediate bridging format. The need to combine both formats to use the knowledge from a multitude of databases in various applications becomes more and more urgent.

In this article, we present a webtool for the translation from BioPAX into SBML format. We demonstrate its functionality by converting the whole PID from BioPAX Level 2 and Level 3 formats to the SBML format, including the qual extension.

## 2 MATERIALS AND METHODS

### 2.1 SBML and the qualitative models extension

The SBML Level 3 Version 1 core specification defines a special XML dialect to describe quantitative models. The most important classes are species, describing reactive species, and reactions, which interconnect species elements. A species element can be further specified with the aid of MIRIAM annotations (Novère *et al.*, 2005). The SBML core specification provides several constructs to describe quantitative processes, such as events, rules, constraints and reactions, but there is no possibility to define qualitative relationships.

The SBML Qualitative Models extension (qual) introduces qualitative elements, such as qualitativeSpecies and transition, providing the necessary means to describe relationships that cannot be described by reactions, for instance, enzyme–enzyme relations or interactions of transcription factors and genes (Berenguier *et al.*, 2011). Instead of the quantities associated to species, which are affected by reactions, qualitativeSpecies exhibit discrete states, representing their activities that are changed using transitions. These transitions are linked to input and output elements. The sign attribute of the input elements describes whether the relationship between the input and output elements is *positive*, *negative*, *dual* or *unknown*. *Dual* means that the transition can operate both activating (*positive*) and inhibiting (*negative*). In contrast, *unknown* is assigned to the input if the transition effect is not further specified. If, in a qualitative model, the activity of Protein A inhibits the activity of Protein B, this would be represented as a transition with an input A, whose sign attribute is *negative*, and an output B.

### 2.2 The BioPAX specification

**Fig. 1.**
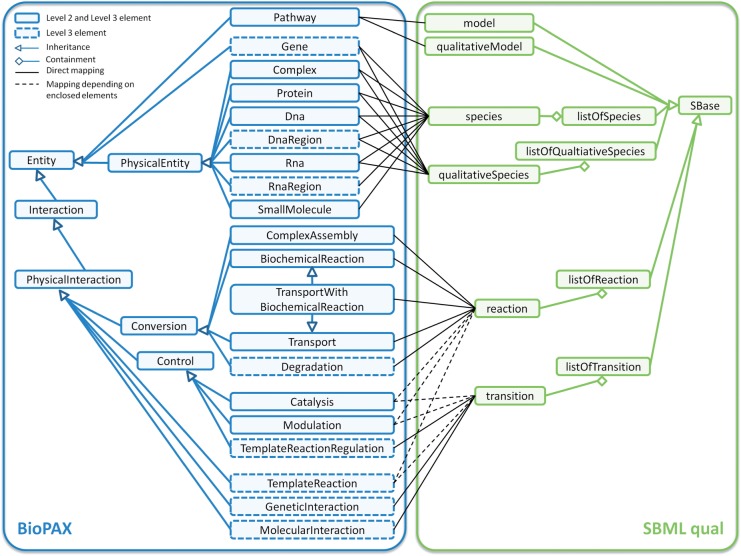
Conversion from BioPAX Level 2 and Level 3 to SBML Level 3 Version 1 with the Qualitative Models extension (qual). The green rounded rectangles on the right-hand side describe the SBML and qual classes, and the blue ones on the left the BioPAX elements. The distinction between BioPAX Level 2 and Level 3 elements is visualized with dashed rectangles. The dashed rectangles denote elements, which are only available in Level 3. All other elements occur in both levels. The ancestry of both BioPAX and SBML elements is indicated with arrows. Lines, ending with a diamond, indicate elements that are contained in other elements. The conversion from BioPAX to SBML qual is drawn with black lines. For some BioPAX elements, it depends on the enclosed entities if the BioPAX element is translated into a reaction or to a relation. This translation dependency is visualized with black dashed lines. A detailed translation description of those elements is shown in Table 2

The BioPAX is a web ontology language (OWL) dialect based on RDF. There is one superclass called Entity that is extended by all other BioPAX classes. Two main classes are distinguished: PhysicalEntity and Interaction. PhysicalEntity describes molecules, such as proteins, complexes, small molecules, DNA or RNA, whereas Interaction defines reactions and relations between PhysicalEntity classes. Interaction is split into Control and Conversion, which can be separated in several subclasses (see Fig. 1).

BioPAX is released level-wise with the current level being Level 3. Level 1 is exclusively able to describe metabolic interactions, whereas Level 2 supports signaling pathways and molecular interactions. In addition to Level 2, gene-regulatory networks and genetic interactions can be described with Level 3. For this purpose, several new BioPAX instances have been added (see dashed elements in Fig. 1). Level 3 is not downwards compatible with Level 2, but Level 2 is downwards compatible with Level 1 (Demir *et al.*, 2010). The BioPAX specification of Level 2 denotes all classes in lower case typewriter font and the specification of Level 3 denotes them in upper case typewriter font. For better readability of this article, all BioPAX element names begin with capital letters and refer to Levels 2 and 3. In contrast, SBML classes are written in lower case.

### 2.3 Conversion of BioPAX to SBML qual

The complete nature PID has been converted from BioPAX to SBML Level 3 Version 1 including the qualitative models extension (qual). PID provides curated pathways from the National Cancer Institute (L2/L3 2012-03-16), pathways from BioCarta (L2 2009-09-09, L3 2010-08-10) and human Reactome pathways (L2/L3 2010-08-10 Schaefer *et al.*, 2009). The translation of the BioPAX Level 2 and Level 3 pathway files is performed in four steps: (i) initializing the models, (ii) translation of PhysicalEntity elements, (iii) translation of Interaction elements and (iv) annotation of all species. An overview of the mapping from BioPAX elements to SBML and to SBML qual elements is shown in Figure 1.

#### 2.3.1 Step 1: initializing the models

First, the pathway organism is determined by searching for the BioSource reference in the BioPAX file. Furthermore, the SBML model and qualitativeModel are built. Both models correspond to the complete pathway represented in the BioPAX file.

#### 2.3.2 Step 2: translation of *PhysicalEntity* elements

Each BioPAX Entity is converted to an SBML species and qualitativeSpecies. In BioPAX, one can specify the nature of the real entity by classes that are derived from Entity (e.g. DNA and Protein). SBML does not contain specific entities that can be derived from an SBML species. The common way to separate different genomic entities in SBML is using SBO terms from the material entity branch. This table specifies the SBO terms that we used to distinguish between various cellular entities in SBML.

In this step, an SBML species and qualitativeSpecies are created for each PhysicalEntity. Depending on the kind of the PhysicalEntity, i.e. if it is a protein, complex, DNA, RNA or small molecule, the species is annotated with the corresponding SBO term (Courtot *et al.*, 2011). The used SBO terms are listed in Table 1. Furthermore, the species compartment is assigned due to the CellularLocation of the PhysicalEntity. The default compartment is set if the CellularLocation is not known.

**Table 1.** BioPAX Entity’s and assigned SBO terms

BioPAX Entity	Assigned SBO term	SBO name
Gene	SBO:0000354	Informational molecule segment
Complex	SBO:0000253	Non-covalent complex
Protein	SBO:0000252	Polypeptide chain
DNA	SBO:0000251	Deoxyribonucleic acid
DnaRegion	SBO:0000251	Deoxyribonucleic acid
Rna	SBO:0000250	Ribonucleic acid
RnaRegion	SBO:0000250	Ribonucleic acid
SmallMolecule	SBO:0000247	Simple chemical

Each BioPAX Entity is converted to an SBML species and qualitativeSpecies. In BioPAX, one can specify the nature of the real entity by classes that are derived from Entity (e.g., DNA, Protein, etc). SBML does not contain specific entities that can be derived from an SBML species. The common way to separate different genomic entities in SBML is using SBO terms from the material entity branch. This table specifies the SBO terms that we used to distinguish between various cellular entities in SBML.

Then, the BioPAX document is mined for an RDF link from the PhysicalEntity to a corresponding Entrez Gene ID. These identifers are unique and facilitate the automated annotation of this species (described in the fourth step). If there exists no Gene ID but a gene symbol, the gene symbol is mapped to a Gene ID.

#### 2.3.3 Step 3: translation of *Interaction* elements

BioPAX Interaction elements are translated into SBML core reactions and qual transitions. An SBML transition describes relationships between molecules that cannot be translated into reactions. Examples for such relationships are enzyme–enzyme relations, protein–protein interactions, interactions of transcription factors and genes, protein–compound interaction, links to other pathways, etc. BioPAX Interaction elements can be split into Conversion and Control elements.

The translation of the Conversion elements is straightforward, because all elements can unambiguously be mapped to SBML reactions. The translation of those elements is performed by creating the same reaction with all substrates, products and enzymes in SBML. Furthermore, the stoichiometry of the reactants and products of BiochemicalReaction and TransportWithBiochemicalReaction is also translated into SBML.

The translation of Control elements is more complicated, because they are translated into a transition or a reaction depending on enclosed Control elements. Control elements always consist of zero or more Controller and zero or one Controlled elements. Controller elements are inherited from PhysicalEntity or Pathway, whereas Controlled elements are also Interaction elements. Thus, it depends on the kind of Controller and Controlled element whether the Interaction is translated into an SBML reaction or transition. All Controller–Controlled combinations and the corresponding SBML classes are listed in Table 2 and discussed in more detail in Supplementary Information. For nearly all Control elements, a ControlType is assigned describing the relationship between the enclosed elements (i.e. activating, inhibiting). Depending on this type, the sign attribute of the SBML input element is determined.

**Table 2.** Description of the translation of BioPAX Control elements

BioPAX Controller	BioPAX Controlled	Converted SBML qual element
**BioPAX Level 3**		
PhysicalEntity	BiochemicalReaction	reaction
PhysicalEntity	ComplexAssembly	reaction
PhysicalEntity	Conversion	transition
PhysicalEntity	Degradation	reaction
PhysicalEntity	Transport	reaction
PhysicalEntity	TransportWithBiochemicalReaction	reaction
PhysicalEntity	Pathway	transition
PhysicalEntity	TemplateReaction	transition
		
Pathway	BiochemicalReaction	transition
Pathway	ComplexAssembly	transition
Pathway	Conversion	transition
Pathway	Degradation	transition
Pathway	Pathway	transition
Pathway	TemplateReaction	transition
Pathway	Transport	transition
Pathway	TransportWithBiochemicalReaction	transition
		
**BioPAX Level 2**		
physicalEntity	biochemicalReaction	reaction
physicalEntity	complexAssembly	reaction
physicalEntity	interaction	transition
physicalEntity	pathway	transition
physicalEntity	transport	reaction
physicalEntity	transportWithBiochemicalReaction	reaction
		
pathway	biochemicalReaction	transition
pathway	complexAssembly	transition
pathway	interaction	transition
pathway	pathway	transition
pathway	transportWithBiochemicalReaction	transition
pathway	tranrtspo	transition

BioPAX Control elements consist of a Controller and one or more Controlled elements. Depending on the kind of Controller or Controlled element, the Control entity is translated into an SBML reaction or transition. The table gives an overview of this conversion regarding BioPAX Level 2 and BioPAX Level 3.

#### 2.3.4 Step 4: annotation of the translated model

Finally, the SBML instances are further annotated. The BioPAX specification allows users to encode arbitrary identifiers for elements. These can be identifiers for various databases, e.g. UniProt, Entrez Gene and Ensembl. Unfortunately, the syntax used in BioPAX is sometimes inconsistent, which leads to XML database annotations like ‘UniProt’ or ‘UniProtKB’ within BioPAX documents that hamper the automatic reading and interpretation of those models by third-party applications.

In SBML, such identifiers can be expressed as standardized MIRIAM URNs that can be added as annotation to any SBML element. We support and add MIRIAM identifiers for the following databases: Entrez Gene, Omim, Ensembl, UniProt, ChEBI, DrugBank, Gene Ontology, HGNC, PubChem, 3DMET, NCBI Taxonomy, PDBeChem, GlycomeDB, LipidBank, EC-Numbers (enzyme nomenclature) and various KEGG databases (gene, glycan, reaction, compound, drug, pathway and orthology). To obtain identifiers for those databases, we map the Entrez Gene identifier, which we annotated on every element in Step 2, to a KEGG identifier. Using the KEGG API, we then query all of those identifiers to retrieve more descriptive names, descriptions of the elements and the mentioned database identifiers. The goal of those annotations is to provide models whose components can uniquely be identified by any application and be linked to external data sources.

BioPAX Control elements consist of a Controller and one or more Controlled elements. Depending on the kind of Controller or Controlled element, the Control entity is translated into an SBML reaction or transition. The table gives an overview of this conversion regarding BioPAX Level 2 and BioPAX Level 3.

### 2.4 Implementation

The conversion was implemented in Java^TM^, using JSBML (Dräger *et al.*, 2011) with the Qualitative Models extension, PaxTools (Demir *et al.*, 2010) and the KEGG API (Kanehisa *et al.*, 2006). PaxTools was used to read the BioPAX files and to manipulate the information content. With the aid of the KEGG API, this information was extended with MIRIAM identifiers (Novère *et al.*, 2005) from the various databases, for instance Entrez Gene, Ensembl and UniProt.

## 3 RESULTS AND DISCUSSION

The PID is a curated and peer-reviewed pathway database containing human pathways with molecular signaling and regulatory events provided by the Nature Cancer Institute, BioCarta, and Reactome. All pathways are provided in XML, BioPAX Level 2 and Level 3 format.

The BioPAX format is perfectly suitable to encode pathway relations and reactions that can be further used for visualization or pathway analysis. However, this format also has its limitations. Many applications, especially for simulation and modeling of biological networks, use the SBML format (Funahashi *et al.*, 2007; Hoops *et al.*, 2006; Zinovyev *et al.*, 2008). Therefore, a few importers and converters for BioPAX into SBML have been developed. BioPAX Entity elements, which can be genes, proteins, small molecules, etc., can be translated into SBML species and the type of the BioPAX Entity can be encoded as SBO term or MIRIAM annotation of the species itself. Relations between Entity elements, corresponding to edges in a pathway graph, are also provided with detailed information in BioPAX. These relations can be transports, biochemical reactions, complex assemblies, etc. At this point, most translations to SBML usually produce errors or have a massive loss of information because the SBML core specification only provides reactions, which represents real biochemical reactions with substrates, products and enzymes. Processes, such as modulation of an entity by another one, cannot directly be encoded as a reaction, at least not without knowing the exact chemical equation. Hence, former conversion approaches from BioPAX to SBML did either incorrectly convert those relations to reactions or simply removed them during the translation. To fill this gap, the SBML community has recently developed the qual specification, which allows users to model arbitrary transitions between species.

Furthermore, the models themselves just provide the base for further analysis or visualization methods. Other applications, such as Clandestine (Funahashi *et al.*, 2007) or COPASI (Hoops *et al.*, 2006), focus on visualization, simulation, analysis, etc. of those models. Therefore, most of those applications have certain requirements on the models. For example, to uniquely map mass spectrometry data on a model, it may be required for the model to have UniProt IDs. To match mRNA expression data or perform gene set enrichment analyses, Entrez Gene identifiers might be required. Consequently, we provide all annotations that we could gather from the input BioPAX files also in the SBML files and further annotate all species with a plethora of additional identifiers.

The qual extension has been created recently and, thus, might not be supported by all applications, yet. Therefore, we decided to build joint SBML core and qual models. All our SBML files contain a model that corresponds to the SBML core specification and an additional qualitativeModel that contains all relations. These models are compatible with older applications that do not yet support qual but still can read all species and reactions. Newer applications that are ready to handle relations can read the additional qual model and process all information that was also available in the BioPAX file.

We converted both the Level 2 and the Level 3 BioPAX files to SBML core, including the qual extension. The reason for converting both levels was the additional description possibility of gene-regulatory networks and genetic interactions in BioPAX Level 3, which is not supported by Level 2 pathway models. Since older simulation applications still work with BioPAX Level 2, we also translated these files into SBML in order to prevent loss of information and to be able to use these models, too. All models are available at http://www.cogsys.cs.uni-tuebingen.de/downloads/Qualitative-Models/. Furthermore, we provide our webtool BioPAX2SBML for further BioPAX translations at http://webservices.cs.uni-tuebingen.de/.

### 3.1 Comparison to other BioPAX to SBML converters

Only a few approaches exist to convert BioPAX to SBML and the existing ones use a simple one-to-one conversion without augmenting the file content for further modeling steps. This might be due to the fact that ‘the inter-conversion between BioPAX and SBML is not trivial as both formats were developed for different purposes’ (Bauer-Mehren *et al.*, 2009). Sybill (Rübenacker *et al.*, 2009) and BiNoM (Zinovyev *et al.*, 2008) are two approaches that can be used for the translation of BioPAX into SBML but none of them is able to appropriately translate signaling networks. Sybill is a stand-alone tool that is also integrated in the quantitative modeling environment VCell (Slepchenko *et al.*, 2003). In contrast, BiNoM is a Cytoscape plugin (Smoot *et al.*, 2011), which offers the possibility to open BioPAX Level 3 files. It mainly focuses on the visualization of BioPAX files and not on the actual conversion into SBML. Table 3 compares these programs based on defined criteria.

Sybill converts BioPAX Level 2 and Level 3 files and has a very comfortable graphical user interface allowing the user to manipulate the conversion result. Unfortunately, the converted SBML files are not complete and the validator from sbml.org reports errors, because species involved in several reactions are missing in the listOfSpecies. Additionally, some groups and pathway links are missing, too. BiNoM generates a complete conversion result, but the validator also reports errors due to empty listOf elements and due to the wrong order of these elements. In contrast to Sybill, BiNoM converts BioPAX files without a pathway element, but is only able to handle a small number of BioPAX files from the PID. Another feature of BiNoM is that it can separately visualize reaction networks, pathway structure and protein–protein interaction networks out of one BioPAX file.

All approaches avoid the translation of duplicate species. Only BioPAX2SBML converter uses the qual extension and SBO terms for detailed species description, translates the BioPAX cross-references (Xrefs) into SBML CV terms and augments the SBML file content with further database cross-references.

## 4 CONCLUSION

Conversion between different formats is important in all parts of computer science. In many cases, conversion leads to errors or a loss of information. The BioPAX to SBML conversion is such an example. Due to limitations of the SBML core specification, it was not possible to include all relationships between reactive species from BioPAX files in SBML files, while producing correct SBML code. But with SBML Level 3 Version 1 and the addition of extensions to the specifications, in particular the qualitative models extension (qual), it is now possible to create accurate and specification-conform SBML code. Using this extension, we produced error-free SBML models while minimizing or even eliminating the loss of information during the translation.

The SBML models, provided along with this publication, consist of SBML species and, wherever possible, exact reaction equations. All relations from the BioPAX documents that could not be converted to exact reactions have been included as qualitative transitions between qualitative species. Additional information, such as various identifiers or the type of an entity, are encoded as SBO terms or MIRIAM URNs of the corresponding elements. By utilizing the KEGG API, it was even possible to complement the translated BioPAX documents with a wealth of information from further databases, such as Entrez Gene and KEGG.

Compared to existing conversion approaches with similar scope, BioPAX2SBML conversions result in comprehensive and correct SBML models, created for all pathways in the nature PID. These models can easily be used, e.g., for further simulation and modeling steps, without having to deal with incorrect input file formats or error-prone conversions.[Table bts508-T3]

**Table 3. bts508-T3:** Comparison of different available converters for BioPAX pathways

	BioPAX2SBML	Sybill	BiNoM
Authors	Büchel *et al.*	Rüebenacker *et al.*	Zinovyev *et al.*
Version	1.0	1.0 (Build 119)	2.0
Release date	2012-04-02	2010-02-11	2012-04-12
BioPAX input level	Levels 2 and 3	Levels 2 and 3	Level 3
SBML Output Level	Level 3 Version 1	Level 2 Version 4	Level 2 Version 4, Beta
			Version for Level 3
Graphical user interface	*✓*	*✓*	*✓*
Qualitative model support	*✓*	−	−
Valid SBML	*✓*	−	−
Complete	*✓*	−	*✓*
No duplicate entities	*✓*	*✓*	*✓*
Robustness	*✓*	○	○
Compartments	*✓*	*✓*	*✓*
Stoichiometry	*✓*	−	−
SBO terms	*✓*	−	−
Xrefs converted into CV terms	*✓*	−	−
Augment model	*✓*	−	−
Provenance	*✓*	−	−

This table compares three applications that are able to translate BioPAX into SBML. The checkmark (*✓*) indicates that the criterion is completely fulfilled, the circle (○) shows that the criterion is partially fulfilled and the minus (−) is used if it is not fulfilled. A conversion is *valid* if the validator from sbml.org reports no errors in the converted SBML file and the converted model is *complete* if no BioPAX entity is missing. The *No duplicate entities* criterion is important for modeling purposes to guarantee that a species is only mentioned once. A converter is *robust* if it can handle all tested files from the Pathway Interaction Database and is able to convert a BioPAX file, which contains no Pathway element. The *Compartments*, *Stoichiometry*, and *Uses SBO terms* criteria denote if this information is translated into SBML and if the SBML species are denoted with the corresponding SBO term. Additionally, it is checked if the BioPAX cross-references (Xrefs) are translated into SBML controlled vocabulary terms (CV terms) and if the SBML model is *augmented* with further information, such as Entrez Gene IDs. Finally, the *provenance* criterion denotes if the file history and conversion tool information is saved in the converted SBML file.

## Supplementary Material

Supplementary Data
