# Supplementary material for: Qualitative translation of relations from BioPAX to SBML qual
Source: Bioinformatics. 2012 Aug 24;28(20):2648–53. doi: 10.1093/bioinformatics/bts508 (PMC3467751; doi:10.1093/bioinformatics/bts508)
Supplement: Supplementary Data [file supp_28_20_2648__index.html]

Qualitative translation of relations from BioPAX to SBML qual — Supplementary Data 

# Qualitative translation of relations from BioPAX to SBML qual

## Supplementary Data

files

**Files in this Data Supplement:**

- Supplementary Data - pdf file
