# Supplementary material for: Qualitative translation of relations from BioPAX to SBML qual
Source: Bioinformatics. 2012 Aug 24;28(20):2648–53. doi: 10.1093/bioinformatics/bts508 (PMC3467751; doi:10.1093/bioinformatics/bts508)
Supplement: Supplementary Data [file supp_bts508_Buechel_Supplement.pdf]

Supplementary information for

Qualitative translation of relations from BioPAX to SBML qual

Finja Büchel[1], Clemens Wrzodek[1], Florian Mittag[1], Andreas Dräger[1], Johannes Eichner[1], Nicolas Rodriguez[2], Nicolas Le Novère[2], and Andreas Zell[1]

1 Center for Bioinformatics Tuebingen (ZBIT), University of Tuebingen, Tübingen, Germany

2 Computational Systems Neurobiology Group, European Bioinformatics Institute, Hinxton, United Kingdom

Detailed description of the conversion of the BioPAX Control element

A `Control` element consists of zero or more `Controller` elements and of zero or one `Controlled` elements. Depending on these enclosed elements a `Control` element can be translated into a SBML transition or reaction. If the `Controller` or the `Controlled` element is a `Pathway` element, the `Interaction` is always converted to a `transition`, because it is biologically not possible to create a reaction with a whole pathway as a reactant or product. A `Conversion` is translated into a `transition` if the `Controlled` element is translated into a `transition`, too. For instance, the conversion of a `Modulation`, consisting of a `PhysicalEntity` as `Controller` and a `BiochemicalReaction` as `Controlled`, is translated into a reaction. An example is shown in Figure S1, which shows the ceramide signaling pathway, where the biochemical reaction from sphingomyelin to ceramide (the `Controlled` element) is positively modulated from SMPD1+ (the `Controller`). This modulation will be converted into a `reaction` where SMPD1+ is modeled as an enzyme of the reaction. But if the `Controlled` element is a `GeneticInteraction`, the `Modulation` is converted into a transition. A detailed overview of the conversion of the `Control` elements is shown in Table 2 of the manuscript. The `sign` attribute of the `input` element describes the relationship between `input` and `output` and is determined depending on the `ControlType` attribute. This attribute is assigned to nearly all `Control` elements. If the `ControlType` is activating, `sign` is set to *active*; if it is inhibiting `sign` is set to *negative*; if it is both, `sign` is set to *dual*; otherwise `sign` is set to *unknown*.

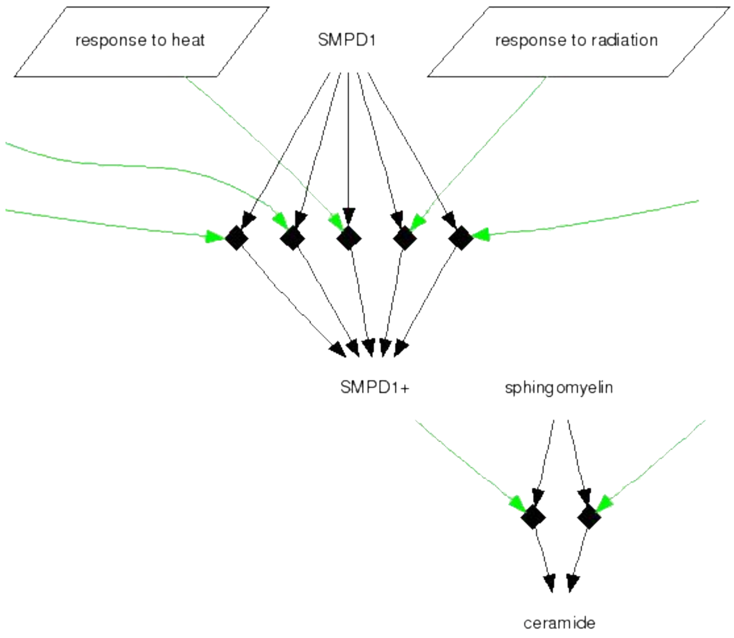

Figure S1: Part of the ceramide signaling pathway imported from BioCarta into the Pathway Interaction Database (PID). Modifications are drawn with black diamonds. The entities with a black arrow to the diamond describe the modulation inputs and the entities with an black arrow out of the diamond the modulation output. The green arrows symbolize positive regulators and the round rectangles complexes. Pathways are visualized with a trapezium. The ceramide signaling pathway is one pathway example for providing information which could not be translated to SBML before the creation of the Qualitative Models extension (qual). With qual, it is possible to translate reactions and relations, and to include them in one model. Even pathway-reaction modulations, like the 'response to heat' pathway that positively stimulates the reaction from SMPD1 to SMPD1+, can be described. (Figure by courtesy of the National Cancer Institute – http://www.cancer.gov).
